# Renal Oncocytoma and Pelvic Urothelial Carcinoma in a Nephrectomy Specimen of a 72-Year-Old Patient

**DOI:** 10.7759/cureus.36061

**Published:** 2023-03-13

**Authors:** Usman Atique, Muhammad Atique, Nadeem B Nusrat, Shujah Muhammad

**Affiliations:** 1 Histopathology, Pakistan Kidney and Liver Institute and Research Center, Lahore, PAK; 2 Urology, Pakistan Kidney and Liver Institute and Research Center, Lahore, PAK

**Keywords:** malignancy, renal, collision, urothelial, oncocytoma

## Abstract

We present the case of a 72-year-old gentleman who presented with a left renal mass. His Computerized Tomography Angiogram showed an 11.8 x 11.3 cm mass involving the upper pole of the left kidney. The mass showed a central stellate scar. There was no locoregional lymphadenopathy. His radical nephrectomy specimen was received in our lab. Sectioning showed a large tumor in the upper pole with a central stellate scar. Microscopically, it showed sheets and nests of round cells with eosinophilic cytoplasm and round nuclei. It was positive for CD117 and negative for CK7. The sections from the renal pelvis showed a urothelial carcinoma arising from the urothelial lining and infiltrating the muscular wall of the renal pelvis. This tumor was positive for CK7 and GATA3. In this case report, we present a rare collision tumor of renal oncocytoma and pelvic urothelial carcinoma.

## Introduction

Oncocytomas are benign epithelial tumors accounting for approximately 5-9% of all renal cell neoplasm with an age range of 24-91 years and peak incidence in the seventh decade of life. The male-to-female ratio is two to one [1]. The majority are asymptomatic and detected on radiology for unrelated symptoms. Radiologically, the most characteristic finding is a central stellate scar; however, it is found in a small proportion of tumors, so radiology alone cannot be used to distinguish it from other renal neoplasms [2]. The characteristic gross appearance is a mahogany-colored or tan mass with a central stellate scar. Microscopically, it is composed of nested architecture with round cells having bland nuclei. Immunohistochemically, they are positive for CD117, S100A1, and E-cadherin while they are negative for CK7, Hale colloidal iron, and vimentin. They may rarely invade the renal sinus fat [3].

Carcinomas of the renal pelvis are rare and constitute about 7% of renal tumors. Most of them are of high-grade urothelial carcinoma histology and present at a higher stage compared to bladder tumors [4]. Other histologic variants could include plasmacytoid, sarcomatoid, micropapillary, and tumors with signet ring change.

On extensive review of the literature, we could find only one reported case of synchronous renal oncocytoma and pelvic urothelial carcinoma [5], although it has been reported with other malignancies also [6,7].

## Case presentation

**Figure 1:**
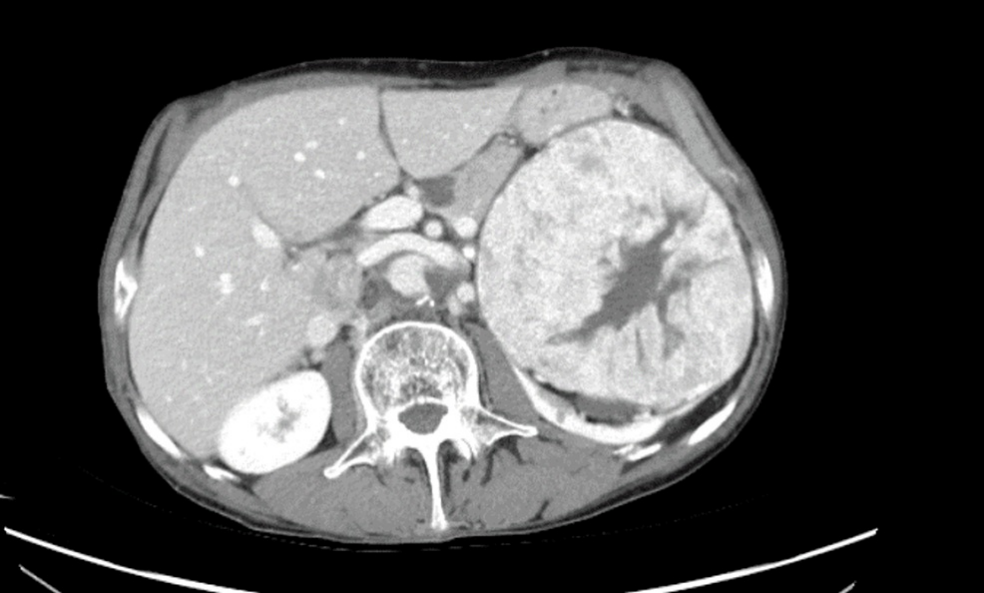
Axial image of CT angiogram showing renal mass with a central stellate scar.

A male, 72 years of age, presented with a history of left urinary tract symptoms with burning micturition and hematuria. On radiologic imaging, he was found to have a left renal mass. His CT renal angiogram showed a vascular mass in the upper pole of the left kidney measuring 11.8 x 11.3 cm with involvement of renal sinus fat and marked splaying of the renal pelvicalyceal system. The mass showed a central area of stellate scar (Figure 1). No locoregional lymphadenopathy or metastatic lung, liver, or bony lesion was noted.

**Figure 2:**
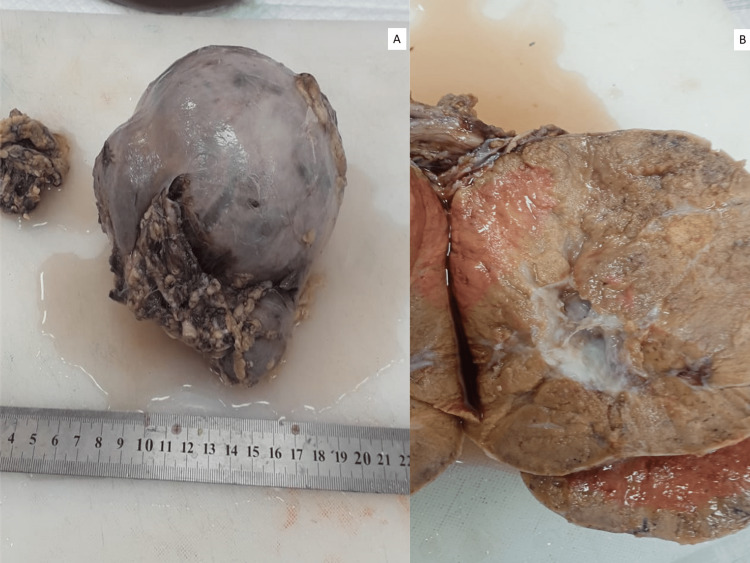
(A) Gross picture of the nephrectomy specimen with tumor in the upper pole. (B) Brown-colored cut surface of the tumor with a central stellate scar.

A nephrectomy specimen was received which showed a large tumor measuring 12 x 7 x 6 cm. The tumor had a brown appearance with a central stellate scar (Figures 2A-2B).

**Figure 3:**
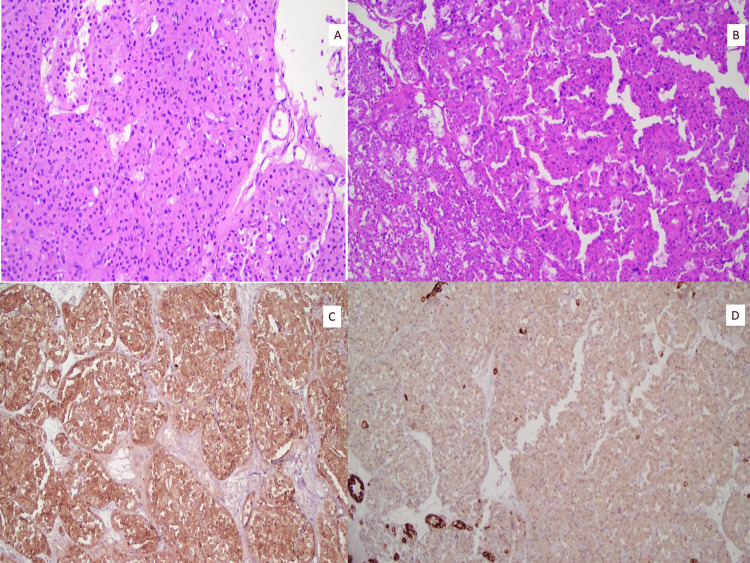
(A-B) Neoplasm composed of cells with eosinophilic cytoplasm and round nuclei arranged as sheets and nests representing oncocytoma. (C) CD117 showing membranous staining in neoplastic cells. (D) CK7 negative in neoplastic cells. Positive in internal control (renal tubules).

The sections from the mass showed a neoplasm composed of sheets and nests of cells with eosinophilic cytoplasm, indistinct cell membranes, and round nuclei. Some areas of degenerative atypia with multinucleation and nuclear enlargement were also seen. The tumor cells were positive for CD117 and negative for CK7, which confirmed this tumor as oncocytoma (Figures 3A-3D). 

**Figure 4:**
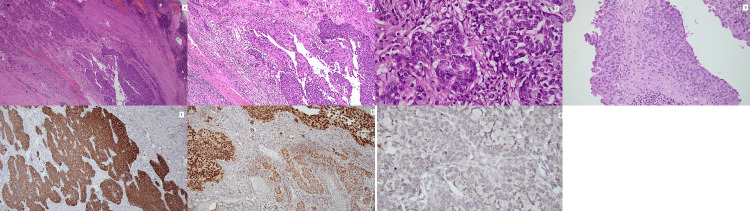
(A) Urothelial carcinoma arising in the pelvic urothelial lining and infiltrating the muscle wall (10x). (B) Urothelial carcinoma arising in the pelvic urothelial lining (20x). (C) Neoplastic cells showing marked nuclear pleomorphism with vesicular nuclei, prominent nucleoli, and atypical mitoses (40x). (D) Carcinoma in situ in ureter (20x). (E) Cytokeratin 7 highlighting the tumor. (F) GATA3 showing nuclear expression in tumor. (G) CD117 negative in tumor.

Sections from the renal pelvis showed a malignant neoplasm composed of infiltrating sheets and cords of atypical cells with vesicular nuclei and prominent nucleoli (Figures 4A-4C). The urothelial lining showed changes in carcinoma in situ in the ureter (Figure 4D). The infiltrative tumor was positive for CK7 (Figure 4E) and GATA 3 (Figure 4F). Unlike the oncocytoma, this tumor was negative for CD117 (Figure 4G). So this was confirmed to be a pelvic urothelial carcinoma infiltrating the muscular wall of the renal pelvis.

Considering the morphology and immunohistochemistry, we gave a diagnosis of dual pathology of renal oncocytoma and pelvic urothelial carcinoma.

## Discussion

Collision tumors are neoplastic lesions composed of two or more distinct cell populations that maintain distinct borders. They are rare but well documented and can be composed of benign and malignant cell populations, two benign populations, or two malignant populations [8]. This term is often used interchangeably with composite tumors; however, collision tumors lack histologic intermingling. Possible hypotheses for this phenomenon include a coincidental occurrence of two tumors, a common carcinogenic stimulus that alters the microenvironment in the vicinity of both the tumors, and finally, the first tumor altering the cellular environment in such a manner as to facilitate the development of a second tumor [9]

Renal oncocytomas are benign renal neoplasms that have been known to coexist with numerous other renal malignancies [6,10,11]. They have a common cell of origin with chromophobe renal cell carcinoma (intercalated cells of the collecting duct). On extensive review of the literature, only one other case of pelvic urothelial carcinoma and renal oncocytoma was found to have been reported [5].

## Conclusions

We present a rare case of collision tumor within the same nephrectomy specimen. Only one case of such a combination was found to be reported in the literature. Renal oncocytomas are benign tumors, but care must be taken not to miss a second malignancy on excision specimens.
